# Density Functional Theory, Molecular Dynamics and AlteQ Studies Approaches of Baimantuoluoamide A and Baimantuoluoamide B to Identify Potential Inhibitors of M^pro^ Proteins: a Novel Target for the Treatment of SARS COVID-19

**DOI:** 10.1134/S0021364023600039

**Published:** 2023-05-15

**Authors:** K. Gurushankar, S. Ch. Jeyaseelan, M. Grishina, I. Siswanto, R. Tiwari, N. N. T. Puspaningsih

**Affiliations:** 1 Laboratory of Computational Modeling of Drugs, Higher Medical and Biological School, South Ural State University, 454080 Chelyabinsk, Russia; 2 Department of Physics, Kalasalingam Academy of Research and Education, 626126 Krishnankoil, Tamilnadu India; 3 Post Graduate & Research Department of Physics, N.M.S.S.V.N. College, 625019 Madurai, Tamilnadu India; 4 Post Graduate Department of Physics, Mannar Thirumalai Naciker College, 625004 Madurai, Tamilnadu India; 5 Bioinformati Laboratory, UCoE Research Center for Bio-Molecule Engineering Universitas Airlangga, 60115 Surabaya, Indonesia; 6 Department of Physics, Coordinator Research and Development Cell, Dr CV Raman University, 495113 Kargi Kota, Bilaspur CG India; 7 Department of Chemistry, Faculty of Science and Technology, Universitas Airlangga, 60115 Surabaya, Indonesia

## Abstract

COVID-19 has resulted in epidemi conditions over the world. Despite efforts by scientists from all over the world to develop an effective va ine against this virus, there is presently no recognized cure for COVID-19. The most succeed treatments for various ailments come from natural components found in medicinal plants, which are also rucial for the development of new medications. This study intends to understand the role of the baimantuoluoamide A and baimantuoluoamide B molecules in the treatment of Covid19. Initially, density functional theory (DFT) used to explore their electronic potentials along with the Becke3-Lee-Yang-Parr (B3LYP) 6-311 + *G*(*d*, *p*) basis set. A number of characteristics, including the energy gap, hardness, local softness, electronegativity, and electrophilicity, have also been calculated to discuss the reactivity of mole ules. Using natural bond orbital, the title compound’s bioactive nature and stability were investigated. Further, both compounds potential inhibitors with main protease (M^pro^) proteins, molecular dynamics simulations and AlteQ investigations also studied.

**Supplementary Information:**

The online version contains supplementary material available at 10.1134/S0021364023600039.

**1. Introduction.** Since the earlier stages of this pandemic, a worldwide effort has been devoted to producing vaccines and antiviral drugs to combat this virus. The novel coronavirus primarily causes severe acute respiratory syndrome (SARS) by interacting with ACE-2 receptors and TMPPRS co-receptors for S protein priming [1–4]. In addition to the primary protease (3CL-pro) and a papain-like protease, it also encodes a cysteine protease, a serine protease (PLpro). These may participate in cleavage of viral polyproteins [5], leading to creation of active substances for replication of virus inside the host cell. One of the most intriguing molecular targets for the development of anti-SARS drugs is the major peptidase ($M_{\rm pro}$) from the coronavirus (CoV) causing severe acute respiratory syndrome (SARS). In this situation, the development of natural medicinal therapies can aid in limiting the virus' transmission [6]. Compared to traditional drug discovery methods which often take a long time, computational approaches provide a convenient and efficient way for discovering new compounds, especially important in the current pandemic. The pharmacological target $3CL_{\rm pro}/M_{\rm pro}$ (PDB ID: 2GTB) is extensively researched for the development of drugs to com bat COVID-19, and is considered one of the most studied targets. This is indicated by the

[1)] Supplementary materials are available for this article at DOI: 10.1134/S0021364023600039 and are accessible for authorized users.

[2)] e-mail: gurushankar01051987@gmail.com abundance of patents and potential drug candidates associated with [7]. It is a proteolytic enzyme required to cleave the viral polyprotein into multiple functionally active protein units. Its selection as a pharmacological target in this study is justified by the fact that its active site is completely conserved and immune to mutations. The major protease 2GTB found in the CoV associated with severe acute respiratory syndrome (SARS), reported that the major protease of 2019-nCov shows 96 % similarity to that of SARS [8].

The interaction between a drug and its target is the most important step of pharmacological action. Drug placement within the receptor pocket is affected by hydrogen bonding, especially short contacts and van der Waals interactions. The proposed approach can be used as an alternative method to determine intermolecular interactions of metal-containing complexes in different systems (enzyme-ligand, reagent-substrate). Molecular regions are determined by all these interactions that ensure ligand-to-receptor complementarity [9]. Recently, various enzyme-ligand complexes (complexes of cyclin-dependent protein kinase, mouse acetylcholinesterase, HIV-1 protease, and EGFR) were studied using the AlteQ method [10–12]. Furthermore, Palko et al., 2021 proposed using AlteQ method to predict biological activity, molecular docking, and study mechanisms of drug action [13]. The AlteQ method uses high-resolution, high-quality, low-temperature X-ray diffraction to describe experimentally determined electron densities. AlteQ is an excellent tool for studying the electronic properties of large molecular structures [10–13]. This method calculates the electron density using Slater's type atomic contributions in the interspace between the receptor and the ligand, and since their interactions are determined by the overlap of electron clouds, they follow the maximum complementarity principle, an equation can be obtained that describes these interactions. This method also evaluates the quality of the interaction between the receptor and the ligand, how complementary the interactions are, and due to this, it is used to reject less realistic structures obtained by docking methods.

Subsequently, de novo drug design is highly time-consuming (usually 10 to 15 years to get new drugs to market). In this regard, either the repurposing of FDA-approved drugs or the search for small molecules from natural consumable sources that are considered safe or have negligible toxicity have been considered appropriate approaches. Computational or in silico techniques represent an exciting approach to the world of drug repurposing. Drug repurposing is an effective approach to drug discovery [14–16]. Recently, it was reported that the CDK4/6 inhibitor palbociclib was one of his top-rated repurposing drugs to treat COVID-19. As a receptor for the development of severe acute respiratory syndrome coronavirus 2 (SARSCoV-2), the expression level of angiotensin-converting enzyme 2 (ACE2) is closely associated with SARS-CoV-2 infection [17]. Previously, our group tested baia and baib alkaloids and concluded that baib has novel structural features and is a highly potent CDK4 inhibitor [18]. However, to our knowledge, neither density functional theory (DFT) studies nor docking simulations have been reported for those compounds. Based on these findings, it seems important to fully understand the two molecules with a view to their potential applications.

In parallel, computational strategies based on density functional theory (DFT), molecular docking, and electronic databases were employed to effectively study and study the pharmacological activities of antiviral drugs against key enzymes of SARS-CoV-2 screening [19–23]. In these studies, DFT provided fundamental insights based on frontier orbital energies and spatial distributions and optimized geometries. This method has helped scientists assess drug stability and calculate structural, electronic, and thermodynamic properties [24]. These properties have been successfully used to better understand drug behavior in biological systems [25, 26]. Frontier molecular orbital energies correlate with computationally and experimentally determined properties of organic molecules screened as drug candidates [27, 28]. Rasool et al. investigated the inhibitory potency of 19 thiazolide derivatives against $M^{pro}$ and methyltransferases. Arafet et al. used a hybrid M06-2X/6-31 + $G(d, p)$:AM1/MM method to investigate inhibition mechanisms of a known $M^{pro}$ inhibitor, N3, and created energy profiles for covalent complex formation with the $M^{pro}$. Khrenova et al. performed a detailed dynamic study of the enzyme-substrate complex of $M^{pro}$. They evaluated the electronic density features of the complex and showed that QM/MM-MD trajectories disclose substrate reactivity in $M^{pro}$, and were in good agreement with relevant experimental data. $M^{pro}$ is a preferred target for SARS-CoV therapeutic design, and a wide range of drug inhibitors have been developed to efficiently target it [29–31]. Previous analyzes of genome sequences have shown that SARS-CoV-2 interacts with corresponding SARS-CoV and MERS-CoV variants with a high degree of sequence similarity [32]. Therefore, $M^{pro}$ is considered a highly promising biological target for SARS-CoV-2.

This article explores DFT, molecular docking, and molecular dynamics simulations with the AlteQ method to investigate the inhibitory potential of screened baia and baib compounds against the major protease ($M^{pro}$)

of the SARS-CoV-2 virus. We first characterize the title compounds in terms of several reactivity parameters using DFT calculations. We then use molecular docking, molecular dynamics simulations and AlteQ together to investigate and analyze binding affinities, binding modes, and interaction stability within the active site of SARS-CoV-2 $M^{pro}$.

**2. Materials and methods.** Baimantuoluoamide A (baia) and baimantuoluoamide B (baib) amide alcoid compounds have been selected from our previous reports and for further detailed theoretical calculation have been studied in the following manner [18]. Utilizing Mulliken population analysis, Fukui functions were obtained by calculating the single point energies of the N, $N-1$, and $\mathrm{N}+1$ species of the molecule using the same basis set $6\text{-}311+G(d,p)$. The GaussView 05 program was used to display the molecule's frontier molecular orbitals and molecular electrostatic potential surface [33]. The DOS spectrum was prepared using the Gausssum 3 algorithm [34].

**3. Results and discussion.**

*3.1. FMO analysis.* The chemical reactivity, kinetic stability, and chemical softness of the compounds baia and baib are all characteristics that may be explained by the molecule's HOMO-LUMO molecular orbitals. The pictorial illustration of HOMO, LUMO, HOMO-1, LUMO + 1, HOMO-2 and LUMO + 2 of baia and baib molecules are shown in Fig. 1. The positive and negative phase is represented in red and green color, respectively. The plots reveal that the HOMO, LUMO, HOMO-1, LUMO+1, HOMO-2 and LUMO + 2 have predicted the variation in the electron density distribution around the whole molecule. This confirms the presence of intermolecular charge transfer in the title molecules. The global hardness, chemical potential, electrophilicity index, and softness of the baia and baib molecules were determined and are shown in along with other FMOs-related molecular parameters (see Supplementary materials Table S1). In general, HOMO-LUMO related molecular properties are used to predict a drug's tendency to engage in drug-acceptor interactions [35]. The ionization potential values were observed larger when compared to electron affinity values describing the better electron-donating capability of the investigated baia and baib molecule. The global hardness value was found to be 2.248 and 2.214 for baia and baib ligand. The global softness value was calculated as 0.445 and 0.452. The chemical hardness values for baia and baib are greater than their softness values. Therefore, higher chemical hardness denotes lower reactivity and higher stability. The chemical potential values baia ($\mu=-3.840$) and baib ($\mu=-3.612$) shows that baib is more stable than baia. The values shown that the baib ligand is more reactive which had strong tendency to form complexes with different metals [33, 34].

*3.2. MEP analysis.* Molecular electrostatic potential (MEP) is a very useful tool for the analysis of the electronic density sites by analysing electrophilic and nucleophilic reactions sites, especially for biomolecules and drugs. It generally provides essential information about the chemical stability and reactivity of an organic molecule to understand the electrophilic and nucleophilic properties. Nucleophilic and electrophilic properties are investigated and mentioned in diferent colors in the region. The blue color indicates higher nucleophilic potential and the red color indicates higher electrophilic potential. The electrostatic potential usually decreases in the order with blue < green < yellow < orange < red. Electrophilic regions are mainly presented around the O atom and the nucleophilic potentials are around the H and N atoms in baia and baib compounds are noticed in Fig. 2 and 3. These reactivity centers enable the compounds to bind with charged systems in the living organism which confirms bioactivity [36].

*3.3. Mulliken atomic charge distribution and local reactivity descriptors.* The calculated Mulliken atomic charge distribution of the molecules baia and baib is shown (see Supplementary materials Tables S2 and S3). The C17 carbon atom in the baia molecule has the largest positive charge because it has connected to the electronegative atoms oxygen (O18) and nitrogen (N19). The hydroxyl group connected to the phenyl ring's oxygen atom (O11) was projected to have the largest negative charge value. All of the hydrogen atoms have positive charge values, while the ring carbon atoms have both positive and negative charge values, indicating that the substituents have a significant impact on the carbon atoms. Calculating Fukui functions is an efficient way to determine how reactive each particular atom in a molecule is. It identifies the preferred regions of a molecule towards the specific chemical events [37, 38]. The molecular reactivity plays an important role on the designing and synthesizing of new pharmaceutical compounds and used to realize the bioactivity of a molecule [39, 40]. The most nucleophilic sites of the baia molecule are in the order of C20 > C24 > C37 > C3 = C30. The most electrophilic sites of the molecule are in the order of C24 > C36 > H23 > C37 > C30. The atoms O18 > H14 > C6 > C4 > H32 are favorable atomic positions for radical attack.

As in the case of baib molecule, C9 carbon atom has the largest positive charge because it attached to the electronegative atoms oxygen (O22) and nitrogen (N20). The nitrogen atom was projected to have the

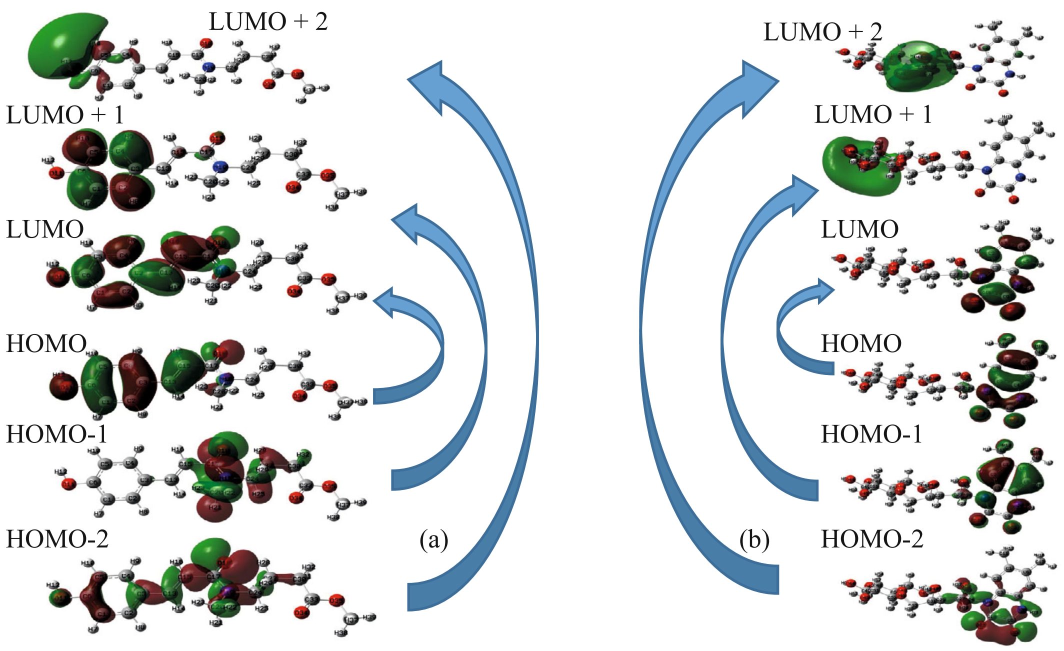

Fig. 1. (Color online) The frontier molecular orbitals of the (a) – baia molecule and (b) – baib molecule

**Table 1.** The average values of trajectory analysis during the simulation time

| Parameters | baia | baib |
|---|---|---|
| Energy total (kcal/mol) | $-89186.80 \pm 200.51$ | $-89076.20 \pm 201.11$ |
| RMSD-complex (nm) | $0.24 \pm 0.02$ | $0.27 \pm 0.02$ |
| RMSD-backbone (nm) | $0.16 \pm 0.03$ | $0.15 \pm 0.02$ |
| RMSD-lligand (nm) | $0.20 \pm 0.03$ | $0.29 \pm 0.07$ |
| RMSF (nm) | $0.46 \pm 0.13$ | $0.76 \pm 0.28$ |
| RoG (nm) | $2.20 \pm 0.01$ | $2.21 \pm 0.00$ |

largest negative charge value (N20). All of the hydrogen atoms have positive charge values, while the ring carbon atoms have both positive and negative charge values, indicating that the substituents have a significant impact on the carbon atoms. The most nucleophilic sites of the baib molecule are in the order of C24 > O43 > O42 > C49 > C52 = C53. The most electrophilic sites of the molecule are in the order of C24 > O42 = H64 > C27 = C53 > C15. The atoms O23 > O22 > H8 > C9 > C10 are favourable atomic sites for radical attack.

*3.4. FMO analysis and density of states (DOS).* Utilizing density of states, it is possible to visualize the molecular orbital (MO) composition and its impact on chemical bonding. DOS spectrum was simulated by convoluting the molecular orbitals with Gaussian curves of unit height. In the DOS spectrum, the occupied and virtual orbitals represented by the green and red lines, respectively. Figure 4 depicts the simulated DOS spectrum of baia and baib.

*3.5. NBO analysis.* The intermolecular and intramolecular interaction, which is defined in terms of stabilization energy $E(2)$, was examined using NBO analysis. The bigger $E(2)$ value, or more electron donating propensity from electron donors to electron acceptors, demonstrates the interaction between electron donors and electron acceptors. The donor-acceptor interactions and associated second order perturbation energies are given in Supplementary materials Tables S4 and S5. The overlap between the $\pi$(C-C) bonding orbital and the $\pi^*$(C-C) anti-bonding orbital produces the hyperconjugative interactions. The intramolecular charge transfer (ICT) of the baia and baib molecules is brought on by this stabilizing interaction. Two strong LP $\rightarrow \pi^*$ interaction in the baia molecule arises due to the lone pairs of the nitrogen and oxygen atoms which stabilize the molecule through LP (2) O35 $\rightarrow \pi^*$C33-O34 and LP (1) N19 $\rightarrow \pi^*$C17-O18 with stabilization energies of 46.53 kJ/mol and 39.01 kcal/mol, respectively. In baib molecule, the strong LP $\rightarrow \sigma^*$ interaction arises

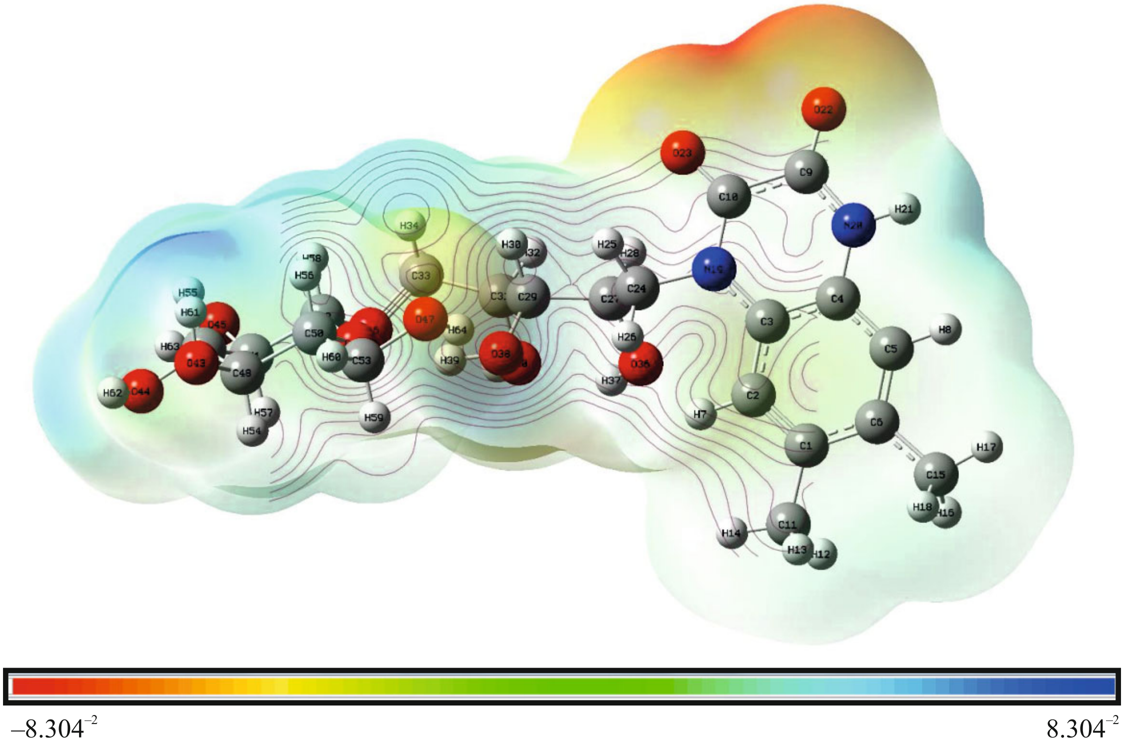

Fig. 2. (Color online) The molecular electrostatic potential surface of the baia molecule

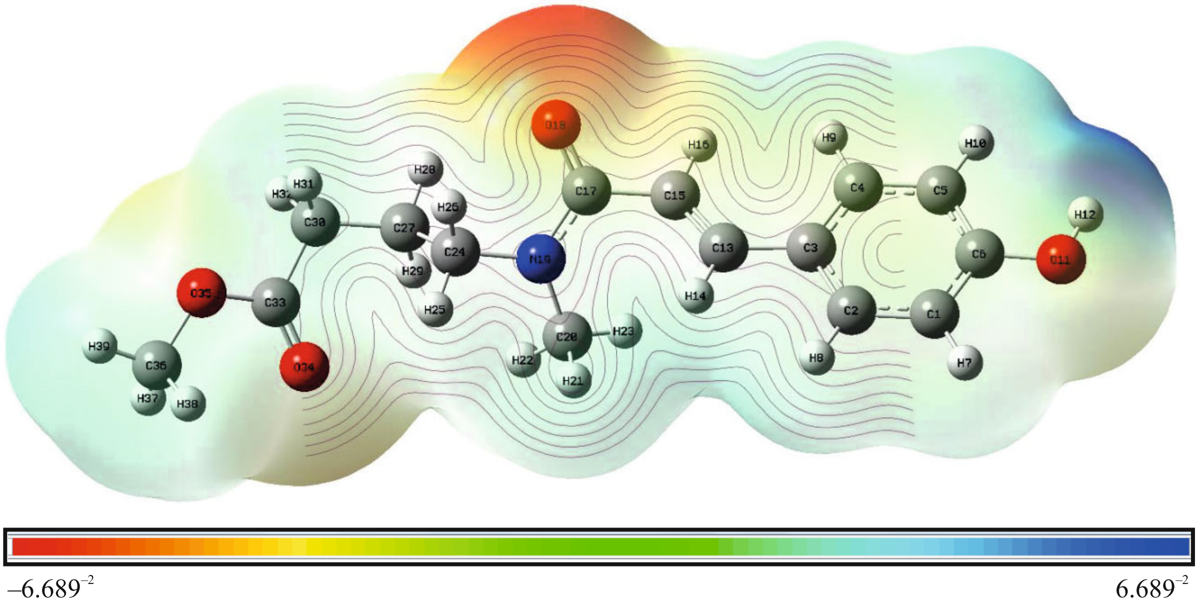

Fig. 3. (Color online) The molecular electrostatic potential surface of the baib molecule due to the lone pairs of the oxygen atoms which stabilize the molecule through LP (2) O38 $\rightarrow$ $\sigma^*$O42-C52, LP (1) O47 $\rightarrow$ $\sigma^*$O42-C52 and LP (1) O47 $\rightarrow$ $\sigma^*$ C53-H60 with stabilization energies of 105.18, 107.49 and 112.04 kcal/mol, respectively. These stabilization interactions between the LP orbitals and $\sigma^*$ orbitals is

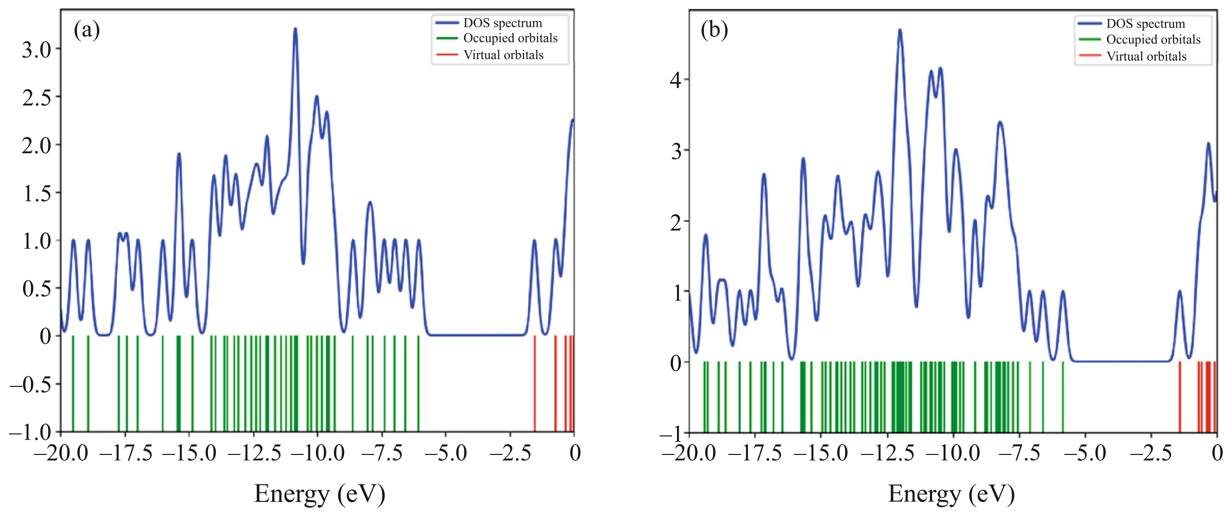

Fig. 4. (Color online) DOS spectrum of the (a) – baia and (b) – baib molecule the characteristic feature of the biological activity of a molecule.

*3.6. Analysis data molecular dynamic simulation.*

*3.6.1. The conformational dynamics of each system: stability, flexibility, and compactness.* The conformational dynamics of each system are evaluated through several variables, such as stability, flexibility, and compactness [41, 42]. The system stability analysis can be done through the convergence of total energy in each system. The results show that there is no excessive energy fluctuation in each system (see Supplementary materials, Fig. S3). Additionally, the calculation of root-mean-square deviation (RMSD) aims to determine the stability of each system in the form of complex, backbone, and ligands (see Supplementary materials, Fig. S4). In particular, the RMSD-complex plays a crucial role in stabilizing the ligand and protein [42]. The results showed that the RMSD-complex did not significantly fluctuate during the 40 ns simulation. It indicates that over time the interaction between the ligand and the receptor has a stable interaction. This assumption is supported by the relatively similar average value of the RMSD-complex baia: $0.24 \pm 0.02$ nm and the baib: $0.27 \pm 0.02$ nm.

In general, RMSF results showed that the ligand target is sufficiently stable, and the conformation has low flexibility. The system flexibility analysis aims to see the flexibility of the protein structure during the simulation time [43]. This variable can be calculated by the root-mean-square fluctuation (RMSF) (Fig. 5). The results show the flexibility of the protein structure of the baib > baia system, it can be seen in the average RMSF value of each system (Table 1). These results identify that the baia system has a more rigid structure than the baib because it has low fluctuations. More specifically, the fluctuations occurred in the loops (216-221) and $\alpha$-helix (256-276) regions. Meanwhile, the residues on the active site (radius of 5 Å from inhibitor coordinate) did not fluctuate excessively. It indicates that the region has a good interaction between inhibitor-amino acids and amino acids-amino acids. Additionally, the results show that each system has a relatively compact structure with insignificant fluctuations ($\sim 2.21$ nm) by calculating the radius of gyration (RoG) (Fig. 5). These results indicate that during the 40 ns simulation time, each system has a well-folded structure [44]. It is confirmed by the average structure over a simulation time of 40 ns (Fig. 5). The results show that both systems have well-folded structures over time compared with crystal structures. The conformational dynamics of each system show good requirements during the simulation process. This becomes a crucial parameter in the evaluation of other variables, such as binding free energy and hydrogen bonding, which will be discussed in the next discussion.

*3.6.2. Hydrogen bonding analysis.* Hydrogen bonding (H-bond) plays a crucial role in inhibitor-protein interactions [45–47]. H-bond analysis was calculated at 40 ns trajectories during the simulation time for each system (Table 2). The analysis of the H-bond occupation plays a major role in the evaluation of inhibitor-protein interactions. The results show that the baia system has four H-bonds and the baib system has only one H-bond is recorded during the simulation time. Unfortunately, the H-bond category shows a weak category because it has a very small H-bond occupancy, which is indicated by the percentage of fraction $< 70\,\%$ [42]. It is because during the simulation time the H-bond was recorded with

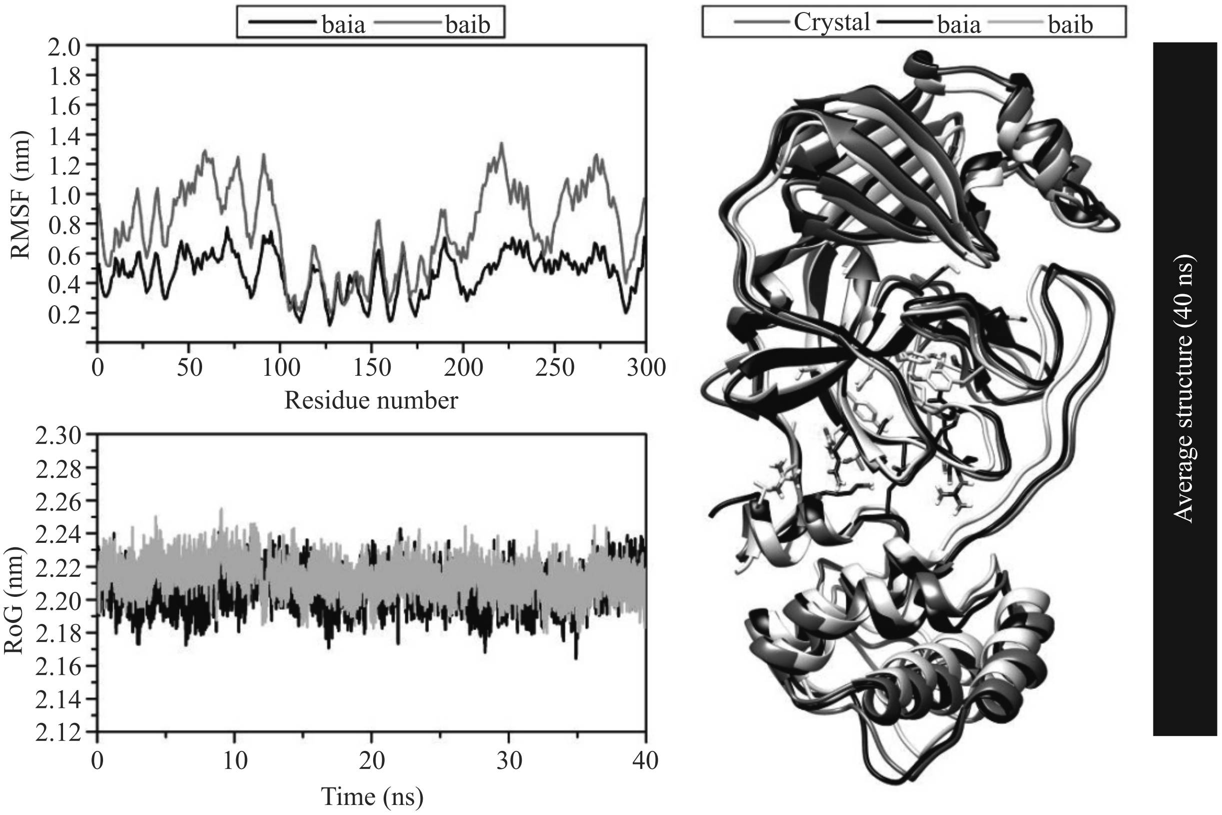

Fig. 5. The root-mean-square of fluctuation and the radius of gyration plotted along 40 ns of trajectories. The superposition showed by the average structure of each system: initial coordinate (crystal) and after simulation along 40 ns (baia and baib)

**Table 2.** Hydrogen bonding analysis using 40 ns trajectories (cutoff value: distance $< 3.5$ Å and angle $> 120°$)

| Contact | Frames | Fraction (%) | Avg-Dist | Avg-Ang |
|---|---|---|---|---|
| baia | | | | |
| O...H-N(Cys144) | 3266 | 16.33 | 3.15 | 153.82 |
| (Hie40)O...H-O1 | 1768 | 8.84 | 3.11 | 154.68 |
| O...H-N(Gly142) | 1716 | 8.58 | 3.13 | 135.32 |
| O...H-N2(Gly182) | 1152 | 5.76 | 3.10 | 146.29 |
| baib | | | | |
| (Lys4)...H-O4 | 1381 | 6.905 | 3.16 | 142.6 |

the highest occupancy presentation only in the low-cost system (Fraction: 16.33 %) of 3266 frames out of 20000 total frames. However, the H-bond analysis provides a fairly clear picture in looking at the type of interaction between inhibitors and proteins.

*3.6.3. Prediction of binding affinity.* Determination of the binding affinity of each inhibitor to the target protein was calculated based on 4000 frames extracted from 40 ns trajectories. The binding affinity calculation process uses the MM-GBSA approach [48, 49]. The calculation process was performed on gas and solvation terms by considering several energy components (Table 3). The calculation process of each energy component becomes the main focus in looking at the contribution of the energy component to the free energy binding ($\Delta G_{\mathrm{bind}}$) [50]. The energy component in the gas phase ($\Delta G_{\mathrm{Gas}}$) shows that the van der Waals energy contribution ($E_{\mathrm{vdW}}$) has a good contribution compared to electrostatic energy ($E_{\mathrm{ele}}$). Besides, the energy contribution in the solvent phase ($\Delta G_{\mathrm{sol}}$) shows an unfavorable contribution to the polar energy ($\Delta G^{\mathrm{eve}}_{\mathrm{solv}}$) for the Generalized Born model. However, overall the $\Delta G_{\mathrm{bind}}$ of each complex showed a good interaction. It is indicated by a higher negative value in thermodynamics terms. It is hoped that inhibitors that have a good binding affinity (higher negative value) can bind strongly to the protein

**Table 3.** Determination of energy component (kcal/mol) of each complex using MM-GBSA approach. Data are shown as mean ± standard error of the mean (SEM)

| Energy component | Baia | Baib |
|---|---|---|
| | Gas term | |
| $E_{\mathrm{vdW}}$ | $-28.84 \pm 0.06$ | $-27.83 \pm 0.08$ |
| $E_{\mathrm{elec}}$ | $-4.50 \pm 0.05$ | $0.00 \pm 0.00$ |
| $\Delta G_{\mathrm{gas}}$ | $-33.34 \pm 0.09$ | $-27.83 \pm 0.08$ |
| | Solvation term | |
| $\Delta G^{\mathrm{ele}}_{\mathrm{solv}}$ | $12.03 \pm 0.05$ | $7.41 \pm 0.02$ |
| $\Delta G^{\mathrm{nonpolar}}_{\mathrm{solv}}$ | $-3.90 \pm 0.00$ | $-3.73 \pm 0.01$ |
| $\Delta G_{\mathrm{sol}}$ | $8.13 \pm 0.04$ | $3.68 \pm 0.01$ |
| | Binding free energy | |
| $\Delta G_{\mathrm{bind(GBSA)}}$ | $-25.21 \pm 0.06$ | $-24.15 \pm 0.07$ |

active site. Each inhibitor that binds to two different active sites shows a relatively similar value (difference: $\sim 1.06$ kcal/mol). These results provide a fairly clear picture of the inhibitor's ability to bind to the target protein.

*3.7. Integration over atomic basins of inhibitor-protein complexes.* Integration over atomic basins of inhibitor-protein complexes was performed using quantum-chemical orbital-free AlteQ approach. This method has recently been shown to describe 3D electron density maps obtained for organic and inorganic compounds using high resolution low temperature X-ray diffraction data with very good quality, superior to other widely known quantum chemical methods [51, 52]. Then various integral molecular and atomic characteristics using AlteQ 3D maps and the Quantum Theory of Atoms in Molecules, or QTAIM, proposed by Bader [53] were computed. We used the software "Electron properties calculation: Integration over atomic basins" available on the site www.chemosophia.com. Radial integration over atomic basins was applied, starting from the atomic center and ending when the electron density surface reaches 0.001 a.u. or when the electron density is equal to the electron density of the neighboring atom. This method is described in more detail in [54]. The most important obtained integral characteristics (molecular solvent-accessible area ($S$), volume ($V$), charge ($Q$), the number of electrons that atoms take from their neighbors ($N_{\mathrm{neigh}}$), the number of electrons which atoms donate to neighbors ($N_{\mathrm{on\,neigh}}$) of the ligands in the gas phase and in the "receptor-ligand" complex as well as their differences ($\Delta$) are given in Table 4.

Integral characteristics show that baia insignificantly transfers electrons to the enzyme in the process of their interaction ($Q > 0$), while baib insignificantly accepts electrons from the enzyme ($Q < 0$). Moreover, the interactions of baib with the enzyme are more effective, which manifests itself in more significant overlaps of the electron clouds of baib with the enzyme: with a constant value of covalent overlaps, intermolecular overlaps are significantly enhanced, which is reflected in a serious increase in $N_{\mathrm{neigh}}$ and $N_{\mathrm{on\,neigh}}$ numbers of electrons, as well as a serious reduction in volume and surface area.

In both cases, interactions are formed both with the transfer of electron density from the ligand to the enzyme, and vice versa. The atomic basins of the ligands in the complexes with the enzyme are shown in Fig. 6. Indeed, the basins of both oxygen atoms and hydrogen atoms of the ligand are truncated. The atoms of the enzyme in contact with them are shown schematically in Fig. 6.

**4. Conclusion.** An alternative strategy that enables the quick identification of prospective therapeutic leads to treat quickly evolving viral infections is the repurposing of pharmaceutical products that have already received approval, including drug prospects. The HOMO and LUMO orbitals and MEP to study intermolecular interaction through the charge transfer within the molecule have been made obvious by DFT. The compounds baia and baib were discovered to be promising leads for the creation of drugs that are selective, safe, and effective against COVID-19, according to DFT studies. Integral characteristic investigations using the AlteQ method indicated that interactions were formed in baia and baib with the transfer of electron density from the ligand to the enzyme and vice versa. Overall, our results propose that baia and baib bearing good binding potency are components of *Datura metel L* that suggest the biologically safe profile of these compounds further supporting the potential of these compounds as starting points for therapeutics against COVID-19. Our studies were performed for identifying bioactive compounds that can inhibit COVID-19 $\mathrm{M^{pro}}$ effectively, as well as providing useful information for future studies. However, further studies should be conducted for the validation of these compounds using *in vitro* and *in vivo* models to pave a way for these compounds in drug discovery.

The work of Dr. K. Gurushankar and Dr. M. Grishina was funded by the Russian Foundation for Basic Research (Russia), Department of Science and Technology (India), and the National Council for Scientific and Technical Development (Brazil) project number 20-53-80002.

**Open Access.** This article is licensed under a Creative Commons Attribution 4.0 International License, which permits use, sharing, adaptation, distribution and

**Table 4.** Integral characteristics ($S$, $V$, $Q$, $N_{\text{neigh}}$, $N_{\text{on neigh}}$) of the ligands in the gas phase and in the "receptor-ligand" complex as well as their differences ($\Delta$)

| Ligands | Parameters | Ligand in gas phase | Ligand in enzyme-ligand complex | $\Delta$ |
|---|---|---|---|---|
| baia | $S$, Å$^2$ | 319.12 | 160.30 | −158.82 |
| | $V$, Å$^3$ | 360.37 | 352.34 | −8.03 |
| | $N_{\text{neigh}}$, $e$ | 19.95987 | 20.34643 | 0.396555 |
| | $N_{\text{on neigh}}$, $e$ | 19.94987 | 20.34734 | 0.397471 |
| | $Q$, $e$ | 0 | 0.00092 | 0.00092 |
| baib | $S$, Å$^2$ | 476.83 | 266.03 | −210.80 |
| | $V$, Å$^3$ | 567.59 | 550.17 | −17.42 |
| | $N_{\text{neigh}}$, $e$ | 32.57773 | 33.17946 | 0.601736 |
| | $N_{\text{on neigh}}$, $e$ | 32.57773 | 33.17544 | 0.597713 |
| | $Q$, $e$ | 0 | −0.00401 | −0.00401 |

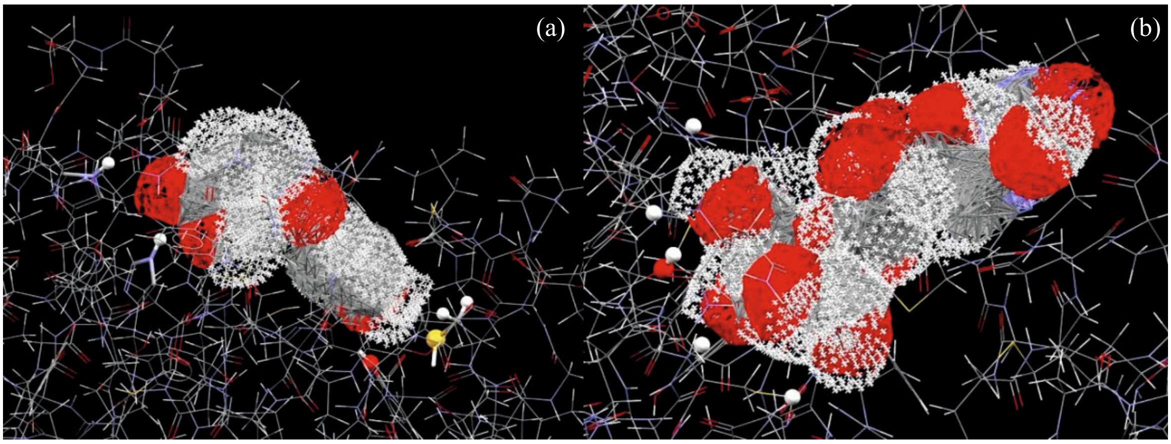

Fig. 6. (Color online) The atomic basins of the ligands in the complexes with the enzyme: (a) – baia; (b) – baib reproduction in any medium or format, as long as you give appropriate credit to the original author(s) and the source, provide a link to the Creative Commons license, and indicate if changes were made. The images or other third party material in this article are included in the article's Creative Commons license, unless indicated otherwise in a credit line to the material. If material is not included in the article's Creative Commons license and your intended use is not permitted by statutory regulation or exceeds the permitted use, you will need to obtain permission directly from the copyright holder. To view a copy of this license, visit http://creativecommons.org/licenses/by/4.0/.

1. S. Boopathi, A. B. Poma, and P. Kolandaivel, J. Biomol. Struct. Dyn. **39**, 3409 (2021).
2. K. Dhama, K. Sharun, R. Tiwari, M. Dadar, Y. S. Malik, K. P. Singh, and W. Chaicumpa, Hum. Vaccin. Immunother. **16**, 1232 (2020).
3. M. Cascella, M. Rajnik, A. Aleem, S. C. Dulebohn, and R. D. Napoli, 2022 Oct. 13. in: StatPearls [Internet]. Treasure Island (FL): StatPearls Publishing (2022).
4. S. Beyersted, E. B. Casaro, and E. B. Range, Eur. J. Clin. Microbiol. Infect. Dis. **40**, 905 (2021).
5. R. Razali, H. Asis, and C. Budiman, Microorganisms **9**, 2481 (2021).
6. S. G. Katre, A. J. Asnani, K. Pratyush, N. G. Sakharkar, A. G. Bhope, K. T. Sawarkar, and V. S. Nimbekar, Futur. J. Pharm. Sci. **8**, 36 (2022).
7. C. Liu, Q. Zhou, Y. Li, L. V. Garner, S. P. Watkins, L. J. Carter, J. Smoot, A. C. Gregg, A. D. Daniels, S. Jervey, and D. Albaiu, ACS Cent. Sci. **25**, 315 (2020).
8. Z. Xu, C. Peng, Y. Shi, Z. Zhu, K. Mu, and X. Wang, https://doi.org/10.1101/2020.01.27.921627 (2020).
9. N. Bohr, Dialectica **2**, 312 (1948).
10. H. Rimac, M. A. Grishina, and V. A. Potemkin, Future Med. Chem. **12**, 1387 (2020).
11. S. Kandagalla, H. Rimac, V. A. Potemkin, and M. A. Grishina, Future Med. Chem. **13**, 863 (2021).
12. H. Rimac, M. Grishina, and V. Potemkin, J. Chem. Inf. Model **61**, 1801 (2021).
13. N. Palko, M. Grishina, and V. Potemkin, Molecules **26**, 3960 (2021).

14. M. Akram, I.M. Tahir, S.M.A. Shah, Z. Mahmood, A. Altaf, K. Ahmad, N. Munir, M. Daniyal, S. Nasir, and H. Mehboob, Phytother. Res. **32**, 811 (2018).
15. Y. Zhao, Y. Wu, and M. Wang, in: *Handbook of Food Chemistry*, P. Cheung and B. Mehta (editors), Springer, Berlin, Heidelberg (2015).
16. V. Kumar, V. Bhatt, and N. Kumar, Studies in Natural Products Chemistry **56**, 287 (2018).
17. Y. Xiao, Y. Yan, L. Chang, H. Ji, H. Sun, S. Song, K. Feng, A. Nuermaimaiti, Z. Lu, and L. Wang, Antiviral Res. **212**, 105558 (2023).
18. K. Gurushankar, H. Rimac, V. Potemkin, and M. Grishina, J. Mol. Struct. **1230**, 129925 (2021).
19. B.R. Beck, B. Shin, Y. Choi, S. Park, and K. Kang, Comput. Struct. Biotechnol. J. **18**, 784 (2020).
20. A.A. Elfiky, Life Sci. **253**, 117592e (2020).
21. A. Belhassan, S. Chtita, H. Zaki, T. Lakhlifi, and M. Bouachrine, Bioinformation **16**, 404 (2020).
22. N. Muralidharan, R. Sakthivel, D. Velmurugan, and M.M. Gromiha, J. Biomol. Struct. Dyn. **39**, 2673 (2021).
23. S. Vardhan and S.K. Sahoo, Comput. Biol. Med. **124**, 103936 (2020).
24. P. Calligari, S. Bobone, G. Ricci, and A. Bocedi, Viruses **12**, 445 (2020).
25. L.S. Braga, D.H. Leal, K. Kuca, and T.C. Ramalho, Curr. Org. Chem. **24**, 314 (2020).
26. R. Sahu, R.K. Mohapatra, S.I. Al-Resayes, D. Das, P.K. Parhi, S. Rahman, L. Pintilie, M. Kumar, M. Azam, and A. Ansari, J. Saudi Chem. Soc. **25**, 101193 (2021).
27. F. Pereira, K. Xiao, D.A. Latino, C. Wu, Q. Zhang, and J. Aires-de-Sousa, J. Chem. Inf. Model **57**, 11 (2017).
28. A. Karuppasamy, K.G. Krishnan, M.P. Velayutham Pillai, and C. Ramalingan, J. Mol. Struct. **1128**, 674 (2017).
29. N. Rasool, F. Yasmin, S. Sahai, W. Hussain, H. Inam, and A. Arshad, Chem. Phys. Lett. **771**, 138463 (2021).
30. M.G. Khrenova, V.G. Tsirelson, and A.V. Nemukhin, Phys. Chem. Chem. Phys. **22**, 19069 (2020).
31. K. Arafet, N. Serrano-Aparicio, A. Lodola, A.J. Mulholland, F.V. González, K. Świderek, and V. Moliner, Chem. Sci. **12**, 1433 (2021).
32. C.M. Coleman, J.M. Sisk, R.M. Mingo, E.A. Nelson, J.M. White, and M.B. Frieman, J. Virol. **90**, 8924 (2016).
33. R. Dennington, T. Keith, and J. Milam, Shawnee Mission KS, 2009. Version 5.
34. N.M. O'Boyle, A.L. Tenderholt, and K.M. Langner, J. Compt. Chem. **29**, 839 (2008).
35. K. Gholivand, F. Mohammadpanah, M. Pooyan, and R. Roohzadeh, Journal of Molecular Structure **1248**, 131481 (2022).
36. H.S. Sumrra, A.U. Hassan, M. Imran, M. Khalid, E.U. Mughal, M.N. Zafar, M.N. Tahir, M.A. Raza, and A.A.C. Braga, Appl. Organomet. Chem. **34**, e5623 (2020).
37. A. Ali, M. Khalid, S. Abid, J. Iqbal, M.N. Tahir, A.R. Raza, J. Zukerman-Schpector, and M.W. Paixao, Appl. Organomet Chem. **34**, e5399 (2020).
38. Y.S. Mary, G. Yalcin, Y.S. Mary, K.S. Resmi, R. Thomas, T. Önkol, E.N. Kasap, and I. Yildiz, Chemical Papers **74**, 1957 (2020).
39. S. Christopher Jeyaseelan and A. Milton Franklin Benial, J. Mol. Recognit. **34**, e2872 (2021).
40. M. Pradeep Kumar, K. Kranthi Raj, D. Ramachandran, M.N.S. Pavan Kumar, Radha Vaddavalli, and P. Jhansi Lakshmi, J. Proteomics Bioinform. **3**, 305 (2010).
41. A. Kumari, V.S. Rajput, P. Nagpal, H. Kukrety, S. Grover, and A. Grover, J. Biomol. Struct. Dyn. **40**, 4987 (2022).
42. B. Nutho, S. Pengthaisong, A. Tankrathok, V.S. Lee, J.R.K. Cairns, T. Rungrotmongkol, and S. Hannongbua, Biomolecules **10**, 1 (2020).
43. A. Bornot, C. Etchebest, and A.G. De Brevern, Proteins Struct. Funct. Bioinforma. **79**, 839 (2011).
44. T. Joshi, T. Joshi, P. Sharma, S. Chandra, and V. Pande, J. Biomol. Struct. Dyn. **39**, 823 (2021).
45. B. Nutho, P. Mahalapbutr, K. Hengphasatporn, N.C. Pattaranggoon, N. Simanon, Y. Shigeta, S. Hannongbua, and T. Rungrotmongkol, Biochemistry **59**, 1769 (2020).
46. R. Suno, S. Lee, S. Maeda, S. Yasuda, K. Yamashita, K. Hirata, S. Horita, M.S. Tawaramoto, H. Tsujimoto, T. Murata, M. Kinoshita, M. Yamamoto, B.K. Kobilka, N. Vaidehi, S. Iwata, and T. Kobayashi, Nat. Chem. Biol. **14**, 1150 (2018).
47. P. Mahalapbutr, N. Darai, W. Panman, A. Opasmahakul, N. Kungwan, S. Hannongbua, and T. Rungrotmongkol, Sci. Rep. **9**, 1 (2019).
48. B.R. Miller, T.D. McGee, J.M. Swails, N. Homeyer, H. Gohlke, and A.E. Roitberg, J. Chem. Theory Comput. **8**, 3314 (2012).
49. C. Wang, D. Greene, L. Xiao, R. Qi, and R. Luo, Front. Mol. Biosci. **4**, 1 (2018).
50. U. Ryde and P. Soderhjelm, Chem. Rev. **116**, 5520 (2016).
51. V.A. Potemkin and M.A. Grishina, J. Comput. Aided Mol. Des. **22**, 489 (2008).
52. M. Grishina, O. Bolshakov, A. Potemkin, and V. Potemkin, Comput. Theor. Chem. **1091**, 122 (2016).
53. R.F.W. Bader, Chem. Rev. **91**, 893 (1991).
54. V. Naumovich, M. Grishina, J. Novak, P. Pathak, V. Potemkin, M. Shahbaaz, and M.H. Abdellattif, J. Biomol. Struct. Dyn. **40**, 4775 (2022).

## Supplementary Information

11448_2023_3598_MOESM1_ESM.pdf
