# Supplementary material for: Density Functional Theory, Molecular Dynamics and AlteQ Studies Approaches of Baimantuoluoamide A and Baimantuoluoamide B to Identify Potential Inhibitors of Mpro Proteins: a Novel Target for the Treatment of SARS COVID-19
Source: JETP Lett. 2023 May 15:1–10. Online ahead of print. doi: 10.1134/S0021364023600039 (PMC10184967; doi:10.1134/S0021364023600039)
Supplement: Supplementary file 1 — 11448_2023_3598_MOESM1_ESM.pdf [file 11448_2023_3598_MOESM1_ESM.pdf]

Supplementary Material to the article

"Density functional theory, molecular dynamics and AlteQ studies approaches of baimantuoluoamide A and baimantuoluoamide B to identify potential inhibitors of M^{pro} proteins: a novel target for the treatment of SARS COVID-19"

Computational resource and data set. The hardware and operating systems utilized were Linux (Intel Core i9-9920X, 64 GB RAM, graphical processing unit (GPU) Nvidia GTX 1080 Ti 11 GB, SSD M.2 250 GB, and SSD SATA 500 GB) and Windows (Intel Core i7 Gen 10 and 16 GB RAM). For preparing the ligands and receptors, Windows-based Chimera version 1.15 with Modeller 9.21 plug-in was used. Molecular dynamic simulation was carried out using the Linux-based programs Gaussian 09W and Amber18 package.

The protein target was obtained from the protein data bank using the PDB code: 2GTB (resolution: 2.00 Å; https://www.rcsb.org/structure/2GTB) [1]. Ligands were obtained from previous docking procedure. The initial coordinate of ligand on protein were obtained from the most stable (the most negative value of the docking score) position of the ligand on the protein.

The simulation findings were significantly influenced by the force fields. During the simulation phase, we employed the ff14SB force field as a part of the AMBER package software. The energy minimization stage, the heating stage, the density stage, the equilibrium stage, and the production stage were among the parameters that were calculated. Leap module was used to design the topology of the ligand, receptor, and ligand-receptor complex. The production stage used in this study was 20 ns to obtain the trajectories needed for analyzing several properties in the molecular dynamic simulation process.

1. T.-W. Lee, M. M. Cherney, J. Liu, K. E. James, J. C. Powers, L. D. Eltis, and M. N. G. James, J. Mol. Biol. **366**, 916 (2007).

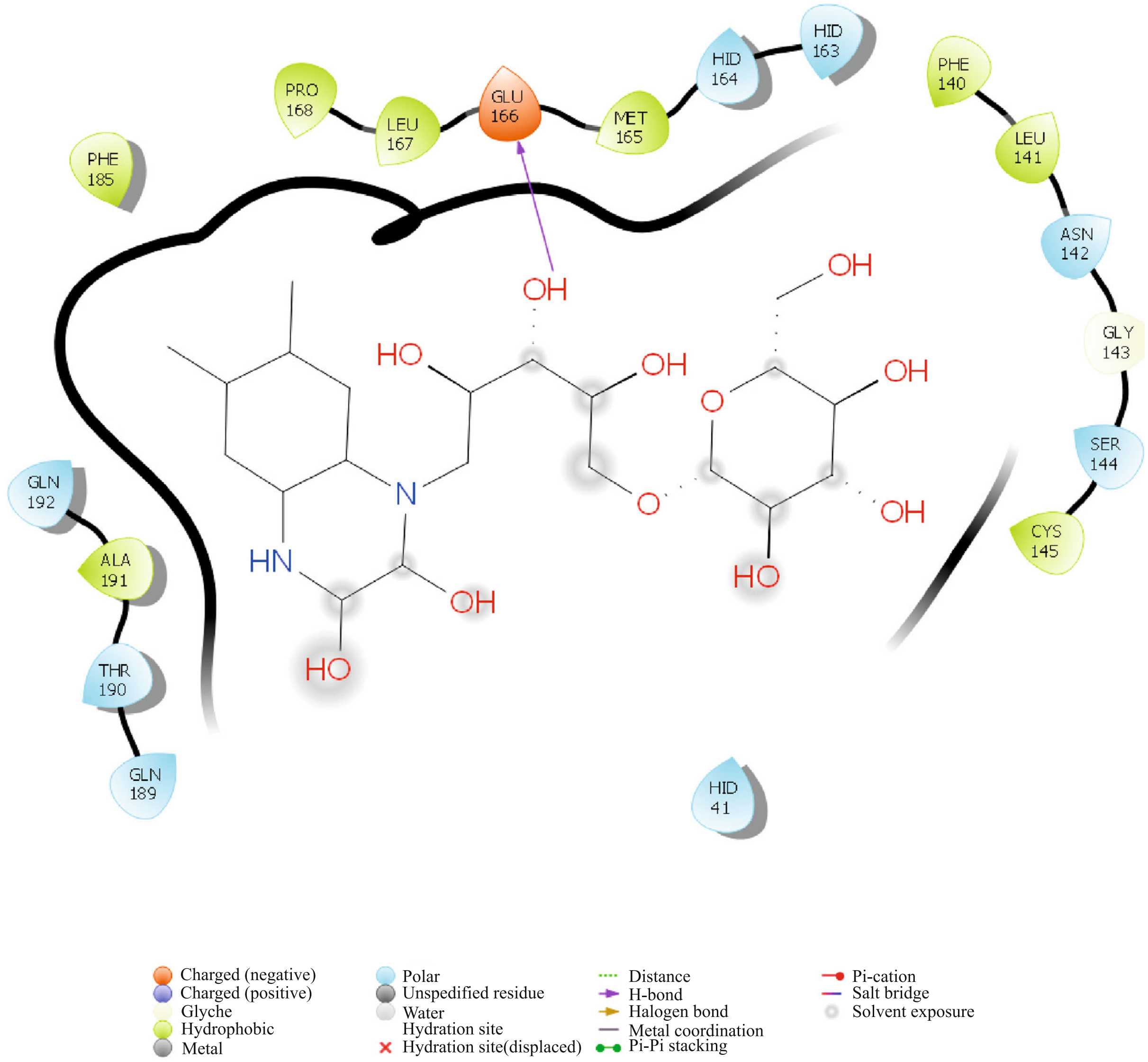

Fig. S1. Docking results of baia

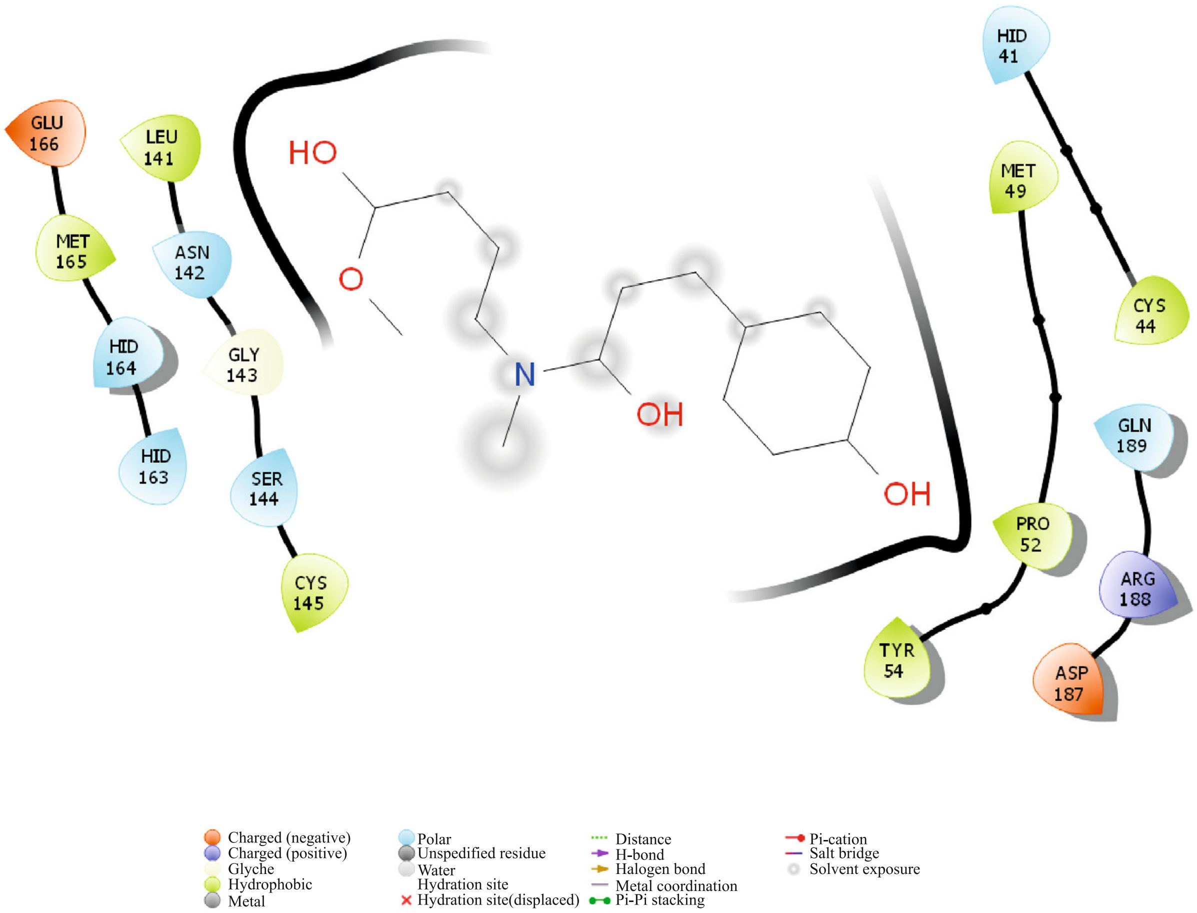

Fig. S2. Docking results of baib

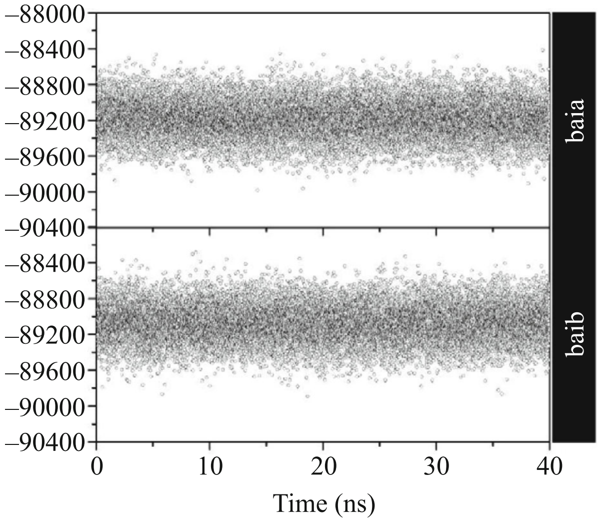

Fig. S3. The mdout analysis plotted along 40 ns of MD simulation

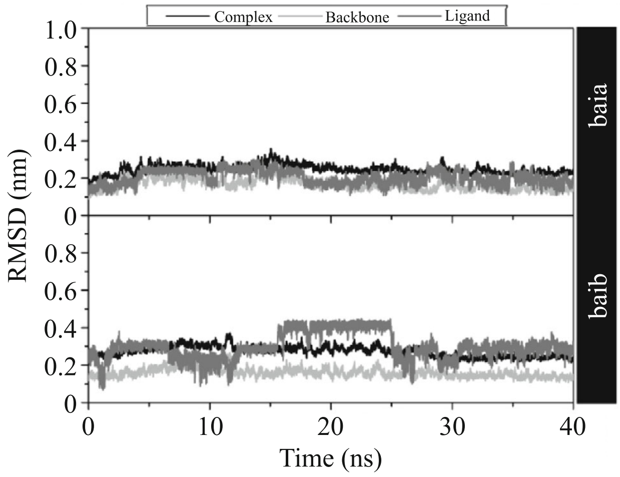

Fig. S4. The root-mean-square displacement of complex, backbone, and ligand for each system plotted along 40 ns of MD simulation

Table S1. The calculated HOMO-LUMO energy values and related molecular properties of the baimantuoluoamide A and baimantuoluoamide B molecule

Molecular properties	Baimantuoluoamide A	Baimantuoluoamide B
	Energy (eV)	Energy (eV)
E_{HOMO} (eV)	−6.089	−5.826
E_{LUMO} (eV)	−1.592	−1.397
Energy gap (eV)	4.497	4.428
Ionization energy (I)	6.089	5.826
Electron affinity (A)	1.592	1.397
Global hardness (η)	2.248	2.214
Chemical potential (μ)	−3.840	−3.612
Global electrophilicity index (ω)	3.280	2.945
Softness (S)	0.445	0.452

Table S2. The calculated Mulliken atomic charges and local reactivity descriptors of the baimantuoluoamide A molecule by B3LYP/6-31 $+G(d,p)$ method

Atom	Mulliken atomic charge values			Fukui Functions		
	Neutral	Cation	Anion	Nucleophilic attack (f_k^+)	Electrophilic attack (f_k^-)	Radical attack (f_k^0)
C1	−0.1074	−0.0757	−0.1240	−0.017	−0.032	0.048
C2	−0.1844	−0.1583	−0.2035	−0.019	−0.026	0.045
C3	0.0964	0.1081	0.1041	0.008	−0.012	0.004
C4	−0.1469	−0.1198	−0.1865	−0.040	−0.027	0.067
C5	−0.1394	−0.1085	−0.1469	−0.007	−0.031	0.038
C6	0.2531	0.2764	0.2070	−0.046	−0.023	0.069
H7	0.1450	0.2153	0.0796	−0.065	−0.070	0.136
H8	0.1308	0.1919	0.0757	−0.055	−0.061	0.116
H9	0.1379	0.1907	0.0896	−0.048	−0.053	0.101
H10	0.1233	0.1916	0.0601	−0.063	−0.068	0.131
O11	−0.6083	−0.5222	−0.6515	−0.043	−0.086	0.129
H12	0.3716	0.4107	0.3421	−0.029	−0.039	0.069
C13	−0.1226	−0.1016	−0.1681	−0.045	−0.021	0.066
H14	0.1335	0.1912	0.0620	−0.071	−0.058	0.129
C15	−0.1510	−0.1003	−0.1995	−0.048	−0.051	0.099
H16	0.1448	0.2032	0.0757	−0.069	−0.058	0.127
C17	0.4514	0.4703	0.4146	−0.037	−0.019	0.056
O18	−0.4672	−0.3752	−0.5382	−0.071	−0.092	0.163
N19	−0.5344	−0.5175	−0.5371	−0.003	−0.017	0.020
C20	−0.2482	−0.2676	−0.2290	0.019	0.019	−0.039
H21	0.1485	0.1796	0.1198	−0.029	−0.031	0.060
H22	0.1644	0.2130	0.1229	−0.042	−0.049	0.090
H23	0.1656	0.1634	0.1708	0.005	0.002	−0.007
C24	−0.1042	−0.1309	−0.0903	0.014	0.027	−0.041
H25	0.1507	0.1839	0.1242	−0.026	−0.033	0.060
H26	0.1696	0.1897	0.1483	−0.021	−0.020	0.041
C37	−0.2304	−0.2404	−0.2180	0.012	0.010	−0.022
H28	0.1623	0.1664	0.1514	−0.011	−0.004	0.015
H29	0.1436	0.1592	0.1282	−0.015	−0.016	0.031
C30	−0.3187	−0.3198	−0.3105	0.008	0.001	−0.009
H31	0.1742	0.1839	0.1560	−0.018	−0.010	0.028
H32	0.1761	0.1981	0.1436	−0.033	−0.022	0.055
C33	0.5103	0.5136	0.4840	−0.026	−0.003	0.030
O34	−0.4250	−0.4237	−0.4458	−0.021	−0.001	0.022
O35	−0.4924	−0.4873	−0.5036	−0.011	−0.005	0.016
C36	−0.1733	−0.1788	−0.1620	0.011	0.005	−0.017
H37	0.1678	0.1771	0.1526	−0.015	−0.009	0.024
H38	0.1678	0.1707	0.1585	−0.009	−0.003	0.012
H39	0.1654	0.1795	0.1436	−0.022	−0.014	0.036

Table S3. The calculated Mulliken atomic charges and local reactivity descriptors of the baimantuoluoamide B molecule by B3LYP/6-31 + $G(d, p)$ method

Atom	Mulliken atomic charge values			Fukui functions		
	Neutral	Cation	Anion	Nucleophilic attack (f_k^+)	Electrophilic attack (f_k^-)	Radical attack (f_k^0)
C1	0.0795	0.0906	0.0779	−0.002	−0.011	0.013
C2	−0.1723	−0.1383	−0.2082	−0.036	−0.034	0.070
C3	0.3565	0.3862	0.3489	−0.008	−0.030	0.037
C4	0.3420	0.3744	0.3432	0.001	−0.032	0.031
C5	−0.1942	−0.1666	−0.2258	−0.032	−0.028	0.059
C6	0.0907	0.1064	0.0885	−0.002	−0.016	0.018
H7	0.1806	0.2267	0.1451	−0.036	−0.046	0.082
H8	0.1243	0.1842	0.0748	−0.050	−0.060	0.109
C9	0.4789	0.5074	0.4017	−0.077	−0.028	0.106
C10	0.4730	0.4965	0.3964	−0.077	−0.024	0.100
C11	−0.4819	−0.4856	−0.4816	0.000	0.004	−0.004
H12	0.1496	0.1910	0.1206	−0.029	−0.041	0.070
H13	0.1475	0.1898	0.1181	−0.029	−0.042	0.072
H14	0.1529	0.1872	0.1344	−0.019	−0.034	0.053
C15	−0.4819	−0.4868	−0.4811	0.001	0.005	−0.006
H16	0.1532	0.1967	0.1243	−0.029	−0.044	0.072
H17	0.1417	0.1796	0.1188	−0.023	−0.038	0.061
H18	0.1525	0.1962	0.1235	−0.029	−0.044	0.073
N19	−0.7184	−0.6864	−0.7186	0.000	−0.032	0.032
N20	−0.7830	−0.7571	−0.7884	−0.005	−0.026	0.031
H21	0.3302	0.3759	0.2817	−0.049	−0.046	0.094
O22	−0.3961	−0.3005	−0.4991	−0.103	−0.096	0.199
O23	−0.4262	−0.3243	−0.5267	−0.101	−0.102	0.202
C24	−0.0943	−0.1253	−0.0749	0.019	0.031	−0.050
H25	0.1783	0.2165	0.1544	−0.024	−0.038	0.062
H26	0.1796	0.2095	0.1489	−0.031	−0.030	0.061
	0.1541	0.1476	0.1569	0.003	0.007	−0.009
H28	0.1426	0.1532	0.1400	−0.003	−0.011	0.013
C29	0.0870	0.0925	0.0830	−0.004	−0.005	0.010
H30	0.1322	0.1400	0.1270	−0.005	−0.008	0.013
C31	0.0911	0.0902	0.0918	0.001	0.001	−0.002
H32	0.1432	0.1457	0.1393	−0.004	−0.002	0.006
C33	−0.0589	−0.0569	−0.0583	0.001	−0.002	0.001
H34	0.1497	0.1536	0.1449	−0.005	−0.004	0.009
H35	0.1634	0.1796	0.1454	−0.018	−0.016	0.034
O36	−0.6298	−0.6321	−0.6265	0.003	0.002	−0.006
H37	0.3951	0.4132	0.3801	−0.015	−0.018	0.033
O38	−0.6377	−0.6398	−0.6362	0.001	0.002	−0.004
H39	0.4184	0.4283	0.4095	−0.009	−0.010	0.019
O40	−0.6105	−0.6138	−0.6081	0.002	0.003	−0.006
H41	0.3791	0.3929	0.3633	−0.016	−0.014	0.030
O42	−0.5684	−0.5769	−0.5614	0.007	0.009	−0.015
O43	−0.5918	−0.5849	−0.5830	0.009	−0.007	−0.002
O44	−0.6156	−0.6092	−0.6218	−0.006	−0.006	0.013
O45	−0.6071	−0.6044	−0.6125	−0.005	−0.003	0.008
O46	−0.4992	−0.4957	−0.5030	−0.004	−0.003	0.007
O47	−0.6192	−0.6188	−0.6192	0.000	0.000	0.000
C48	0.0670	0.0664	0.0702	0.003	0.001	−0.004
C49	0.0819	0.0820	0.0880	0.006	0.000	−0.006
C50	0.0891	0.0871	0.0853	−0.004	0.002	0.002

Atom	Mulliken atomic charge values			Fukui functions		
	Neutral	Cation	Anion	Nucleophilic attack (f_k^+)	Electrophilic attack (f_k^-)	Radical attack (f_k^0)
C51	0.1255	0.1268	0.1220	−0.004	−0.001	0.005
C52	0.3493	0.3437	0.3539	0.005	0.006	−0.010
C53	−0.0233	−0.0300	−0.0186	0.005	0.007	−0.011
H54	0.1767	0.1803	0.1623	−0.014	−0.004	0.018
H55	0.1186	0.1241	0.1070	−0.012	−0.005	0.017
H56	0.1295	0.1345	0.1240	−0.005	−0.005	0.010
H57	0.1638	0.1668	0.1543	−0.009	−0.003	0.012
H58	0.1359	0.1418	0.1269	−0.009	−0.006	0.015
H59	0.1398	0.1427	0.1313	−0.008	−0.003	0.011
H60	0.1645	0.1778	0.1527	−0.012	−0.013	0.025
H61	0.3604	0.3635	0.2883	−0.072	−0.003	0.075
H62	0.3792	0.3847	0.3509	−0.028	−0.006	0.034
H63	0.3838	0.3906	0.3717	−0.012	−0.007	0.019
H64	0.3777	0.3687	0.3815	0.004	0.009	−0.013

Table S4. Second order perturbation theory analysis of Fock matrix of the baimantuoluoamide A molecule by NBO method

Donor (i)	ED (i) e	Acceptor (j)	ED (j) e	$E(2)a$ (Kcal/mol)	$E(i)-E(j)b$ (arb. units)	$F(i,j)c$ (arb. units)
π C 1 – C 6	1.62339	π^* C 2 – C 3	0.39456	23.45	0.29	0.074
π C 1 – C 6		π^* C 4 – C 5	0.32505	17.20	0.28	0.063
π C 2 – C 3	1.62780	π^* C 1 – C 6	0.02480	19.16	0.27	0.064
π C 2 – C 3		π^* C 4 – C 5	0.32505	22.37	0.27	0.07
π C 2 – C 3		π^* C 13 – C 15	0.11125	15.66	0.3	0.066
π C 4 – C 5	1.71565	π^* C 1 – C 6	0.02480	22.03	0.28	0.072
σ C 4 – C 5		σ^* C 2 – C 3	0.39456	16.03	0.29	0.063
π C 13 – H 14	1.98127	π^* C 15 – H 16	0.01701	5.27	0.95	0.063
π C 13 – C 15	1.88982	π^* C 2 – C 3	0.39456	10.99	0.3	0.056
π C 13 – C 15		π^* C 17 – O 18	0.29289	11.72	0.37	0.062
σ C 15 – H 16	1.97070	σ^* C 13 – H 14	0.01771	5.36	0.94	0.063
σ C 30 – H 32	1.95879	σ^* C 33 – O 34	0.22194	6.25	0.51	0.053
LP (1) O 11	1.97954	σ^* C 5 – C 6	0.02741	6.00	1.16	0.075
LP (2) O 11	1.87331	π^* C 1 – C 6	0.38295	26.81	0.35	0.093
LP (2) O 18	1.86209	σ^* C 15 – C 17	0.05511	17.03	0.67	0.098
LP (2) O 18		σ^* C 17 – N 19	0.08082	23.05	0.71	0.116
LP (1) N 19	1.67510	π^* C 17 – O 18	0.29289	39.01	0.34	0.103
LP (1) N 19		σ^* C 20 – H 21	0.01849	6.88	0.63	0.064
LP (1) N 19		σ^* C 24 – C 27	0.02182	6.49	0.63	0.062
LP (2) O 34	1.85042	σ^* C 30 – C 33	0.06074	17.80	0.65	0.098
LP (2) O 34		σ^* C 33 – O 35	0.10151	32.63	0.63	0.13
LP (1) O 35	1.96348	σ^* C 33 – O 34	0.02304	7.36	1.18	0.084
LP (1) O 35		σ^* C 36 – H 37	0.01347	0.55	0.95	0.021
LP (2) O 35	1.79462	π^* C 33 – O 34	0.22194	46.53	0.34	0.113

Table S5. Second order perturbation theory analysis of Fock matrix of the baimantuoluoamide B molecule by NBO method

Donor (i)	ED (i) e	Acceptor (j)	ED (j) e	$E(2)a$ (Kcal/mol)	$E(i){-}E(j)b$ (arb. units)	$F(i,j)c$ (arb. units)
π C 1 – C 2	1.68222	π^* C 3 – C 4	1.62693	20.65	0.27	0.07
π C 1 – C 2		σ^* C 5 – C 6	1.70029	21.61	0.27	0.07
σ C 1 – C 6	1.96819	σ^* C 1 – C 11	1.98083	7.76	1.23	0.09
σ C 1 – C 6		σ^* C 5 – H 8	1.97812	18.40	1.20	0.13
σ C 1 – C 6		σ^* C 24 – H 26	1.97962	5.06	1.21	0.07
σ C 1 – C 6		σ^* O 42 – C 50	1.98389	12.75	1.02	0.10
σ C 1 – C 6		σ^* O 42 – C 52	1.98722	42.27	0.88	0.17
σ C 1 – C 6		σ^* O 47 – H 64	1.98835	1.82	1.27	0.04
σ C 1 – C 6		σ^* C 50 – H 56	1.97539	6.39	1.14	0.08
σ C 1 – C 6		σ^* C 52 – H 58	1.98353	14.34	3.28	0.19
σ C 1 – C 6		σ^* C 53 – H 60	1.97649	15.72	3.85	0.22
σ C 1 – C 11	1.98083	σ^* C 1 – C 2	1.96986	9.16	3.01	0.15
σ C 1 – C 11		σ^* C 1 – C 2	1.68222	6.41	2.46	0.12
σ C 1 – C 11		σ^* C 1 – C 11	1.98083	11.85	2.83	0.16
σ C 1 – C 11		σ^* C 5 – C 6	1.70029	10.95	2.45	0.16
σ C 1 – C 11		σ^* C 5 – H 8	1.97812	40.01	2.80	0.30
σ C 1 – C 11		σ^* C 10 – N 19	1.98643	10.24	2.84	0.16
σ C 1 – C 11		σ^* C 11 – H 12	1.97885	6.51	2.82	0.12
σ C 1 – C 11		σ^* C 33 – H 34	1.98150	21.91	2.75	0.22
σ C 1 – C 11		σ^* C 33 – H 35	1.98233	48.55	2.72	0.33
σ C 1 – C 11		σ^* O 36 – H 37	1.98701	14.12	2.86	0.18
σ C 1 – C 11		σ^* O 42 – C 50	1.98389	59.72	2.61	0.35
σ C 1 – C 11		σ^* O 42 – C 52	1.98722	180.58	2.47	0.60
σ C 1 – C 11		σ^* O 47 – C 53	1.99393	11.99	2.70	0.16
σ C 1 – C 11		* O 47 – H 64	1.98835	9.78	2.86	0.15
σ C 1 – C 11		σ^* C 50 – H 56	1.97539	31.99	2.73	0.27
σ C 1 – C 11		σ^* C 52 – H 58	1.98353	115.80	4.87	0.68
σ C 1 – C 11		σ^* C 53 – H 60	1.97649	135.14	5.45	0.77
σ C 3 – C 4	1.62693	σ^* C 1 – C 2	1.68222	5.32	0.39	0.04
σ C 3 – C 4		σ^* C 33 – H 35	1.98233	8.11	0.66	0.07
σ C 3 – C 4		σ^* O 42 – C 50	1.98389	11.82	0.54	0.08
σ C 3 – C 4		σ^* O 42 – C 52	1.98722	46.85	0.40	0.13
σ C 15 – H 18	1.97867	σ^* O 42 – C 52	1.98722	14.28	0.58	0.08
σ N 19 – C 24	1.98338	σ^* C 1 – C 11	1.98083	12.48	3.32	0.18
σ N 19 – C 24		σ^* C 5 – C 6	1.70029	10.82	2.94	0.18
σ N 19 – C 24		σ^* C 5 – H 8	1.97812	39.55	3.28	0.32
σ N 19 – C 24		σ^* C 24 – H 26	1.97962	24.92	3.29	0.26
σ N 19 – C 24		σ^* C 33 – H 34	1.98150	22.52	3.24	0.24
σ N 19 – C 24		σ^* C 33 – H 35	1.98233	49.74	3.21	0.36
σ N 19 – C 24	1.98338	σ^* O 36 – H 37	1.98701	14.64	3.35	0.20
σ N 19 – C 24		σ^* O 42 – C 50	1.98389	60.80	3.10	0.39
σ N 19 – C 24		σ^* O 42 – C 52	1.98722	182.21	2.96	0.66
σ N 19 – C 24		σ^* O 47 – C 53	1.99393	12.21	3.19	0.18
σ N 19 – C 24		σ^* O 47 – H 64	1.98835	10.03	3.35	0.16
σ N 19 – C 24		σ^* C 50 – H 56	1.97539	32.77	3.22	0.29

Donor (i)	ED (i) e	Acceptor (j)	ED (j) e	$E(2)a$ (Kcal/mol)	$E(i)-E(j)b$ (arb. units)	$F(i,j)c$ (arb. units)
σ N 19 – C 24		σ^* C 52 – H 58	1.98353	123.03	5.36	0.73
σ N 19 – C 24		σ^* C 53 – H 60	1.97649	138.61	5.94	0.81
σ C 24 – C 27	1.97561	σ^* C 5 – H 8	1.97812	21.26	1.52	0.16
σ C 24 – C 27		σ^* C 10 – N 19	1.98643	8.41	1.57	0.10
σ C 24 - C 27		σ^* C 24 - H 26	1.97962	18.56	1.53	0.15
σ C 24 – C 27		σ^* C 33 – H 34	1.98150	12.24	1.48	0.12
σ C 24 – C 27		σ^* C 33 – H 35	1.98233	27.61	1.45	0.18
σ C 24 – C 27		σ^* O 36 – H 37	1.98701	14.65	1.59	0.14
σ C 24 – C 27		σ^* O 42 – C 50	1.98389	34.53	1.34	0.19
σ C 24 – C 27		σ^* O 42 – C 52	1.98722	109.99	1.20	0.33
σ C 24 – C 27		σ^* C 50 – H 56	1.97539	17.82	1.46	0.15
σ C 24 - C 27		σ^* C 52 – H 58	1.98353	53.25	3.60	0.39
σ C 24 – C 27		σ^* C 53 – H 60	1.97649	62.99	4.18	0.46
σ C 29 - H 30	1.96780	σ^* O 42 - C 50	1.98389	10.69	0.79	0.08
σ C 29 – H 30		σ^* O 42 – C 52	1.98722	36.59	0.65	0.14
σ C 29 – O 38	1.99226	σ^* O 42 – C 52	1.98722	9.66	0.89	0.08
σ O 38 – H 39	1.98839	σ^* C 1 – C 11	1.98083	14.83	4.19	0.22
σ O 38 – H 39		σ^* C 5 – C 6	1.70029	11.78	3.81	0.21
σ O 38 – H 39		σ^* C 5 – H 8	1.97812	45.04	4.15	0.39
σ O 38 - H 39		σ^* C 10 - N 19	1.98643	12.24	4.20	0.21
σ O 38 – H 39		σ^* C 24 - H 26	1.97962	29.56	4.16	0.31
σ O 38 – H 39		σ^* C 33 – H 34	1.98150	25.14	4.11	0.29
σ O 38 – H 39		σ^* C 33 – H 35	1.98233	54.25	4.08	0.42
σ O 38 – H 39		σ^* O 36 - H 37	1.98701	15.65	4.22	0.23
σ O 38 – H 39		σ^* O 42 – C 50	1.98389	64.59	3.97	0.46
σ O 38 – H 39		σ^* O 42 – C 52	1.98722	196.62	3.83	0.79
σ O 38 – H 39		σ^* O 47 – C 53	1.99393	13.47	4.06	0.21
σ O 38 – H 39		σ^* C 50 – H 56	1.97539	36.18	4.09	0.35
σ O 38 – H 39		σ^* C 52 – H 58	1.98353	106.02	6.23	0.73
σ O 38 – H 39		σ^* C 53 – H 60	1.97649	105.33	6.81	0.76
σ O 40 – H 41	1.98903	σ^* O 42 – C 52	1.98722	13.32	0.84	0.10
σ O 43 – C 48	1.99067	σ^* C 1 – C 11	1.98083	8.14	2.18	0.12
σ O 43 – C 48		σ^* C 5 – H 8	1.97812	25.78	2.14	0.21
σ O 43 – C 48		σ^* C 24 – H 26	1.97962	16.76	2.15	0.17
σ O 43 – C 48		σ^* C 33 – H 34	1.98150	14.41	2.09	0.16
σ O 43 – C 48	1.99067	σ^* C 33 – H 35	1.98233	32.07	2.07	0.23
σ O 43 – C 48		σ^* O 36 – H 37	1.98701	9.34	2.21	0.13
σ O 43 – C 48		σ^* O 42 – C 50	1.98389	52.29	1.96	0.29
σ O 43 – C 48		σ^* O 42 – C 52	1.98722	123.65	1.82	0.43
σ O 43 – C 48		σ^* O 47 – C 53	1.99393	8.01	2.05	0.11
σ O 43 – C 48		σ^* C 52 – H 58	1.98353	65.26	4.22	0.47
σ O 43 – C 48		σ^* C 53 – H 60	1.97649	78.33	4.80	0.55
σ O 43 – H 61	1.98827	σ^* O 42 – C 52	1.98722	8.63	1 0.83	0.08
σ O 45 – C 51	1.99067	σ^* O 42 – C 52	1.98722	31.15	0.97	0.16
σ O 47 – C 53	1.99393	σ^* C 5 – H 8	1.97812	9.95	1.03	0.09
σ O 47 – C 53		σ^* C 33 – H 35	1.98233	15.03	0.96	0.11
σ O 47 – C 53		σ^* O 42 – C 50	1.98389	15.69	0.84	0.10
σ O 47 – C 53		σ^* O 42 – C 52	1.98722	69.69	0.71	0.20
σ O 47 – C 53		σ^* C 53 – H 60	1.97649	17.52	3.68	0.23

Donor (i)	ED (i) e	Acceptor (j)	ED (j) e	$E(2)a$ (Kcal/mol)	$E(i)-E(j)b$ (arb. units)	$F(i,j)c$ (arb. units)
σ O 47 – H 64	1.98835	σ^* C 5 - H 8	1.97812	26.48	1.01	0.15
σ O 47 – H 64		σ^* C 24 – H 26	1.97962	19.09	1.02	0.13
σ O 47 – H 64		σ^* C 33 – H 34	1.98150	14.95	0.96	0.11
σ O 47 – H 64		σ^* C 33 – H 35	1.98233	35.28	0.94	0.16
σ O 47 – H 64		σ^* O 36 – H 37	1.98701	10.01	1.08	0.09
σ O 47 – H 64		σ^* O 42 – C 50	1.98389	49.16	0.83	0.18
σ O 47 – H 64		σ^* O 42 – C 52	1.98722	170.24	0.69	0.31
σ O 47 – H 64		σ^* O 47 – H 64	1.98835	12.49	1.08	0.10
σ O 47 – H 64		σ^* C 50 – H 56	1.97539	18.94	0.95	0.12
σ O 47 – H 64		σ^* C 52 – H 58	1.98353	64.47	3.09	0.40
σ O 47 – H 64		σ^* C 53 – H 60	1.97649	88.23	3.66	0.51
σ C 53 – H 59	1.98356	σ^* O 42 – C 52	1.98722	12.63	0.57	0.08
σ C 53 – H 59		σ^* C 50 – H 56	1.97539	8.59	0.83	0.08
σ C 53 – H 60	1.97649	σ^* O 42 – C 50	1.98389	14.11	0.72	0.09
σ C 53 - H 60		σ^* O 42 – C 52	1.98722	11.96	0.58	0.08
LP (1) N 19	1.60721	σ^* C 3 – C 4	1.62693	33.99	0.28	0.09
LP (1) N 19		σ^* C 10 – O 23	1.98055	56.60	0.28	0.11
LP (1) N 20	1.63839	σ^* C 3 – C 4	1.62693	35.43	0.30	0.09
LP (1) N 20		σ^* C 9 – O 22	1.99332	56.40	0.29	0.12
LP (2) O 22	1.85338	σ^* C 9 – C 10	1.97339	22.81	0.60	0.11
LP (2) O 22		σ^* C 9 – N 20	1.98961	25.60	0.70	0.12
LP (2) O 23	1.85640	σ^* C 9 – C 10	1.97339	19.74	0.62	0.10
LP (2) O 23	1.85640	σ^* C 10 – N 19	1.98643	31.87	0.68	0.13
LP (2) O 23		σ^* O 42 – C 52	1.98722	10.65	0.32	0.05
LP (2) O 36	1.95133	σ^* C 27 – H 28	1.97089	9.13	0.67	0.07
LP (1) O 38	1.97470	σ^* O 42 – C 52	1.98722	15.60	0.	0.09
LP (2) O 38	1.93679	σ^* C 5 – H 8	1.97812	15.85	0.92	0.11
LP (2) O 38		σ^* C 24 – H 26	1.97962	10.30	0.93	0.09
LP (2) O 38	1.93679	σ^* C 33 – H 34	1.98150	9.02	0.87	0.08
LP (2) O 38		σ^* C 33 – H 35	1.98233	21.21	0.85	0.12
LP (2) O 38		σ^* O 42 – C 50	1.98389	29.48	0.74	0.13
LP (2) O 38		σ^* O 42 – C 52	1.98722	105.18	0.60	0.22
LP (2) O 38		σ^* C 50 – H 56	1.97539	13.76	0.86	0.10
LP (2) O 42		σ^* C 5 – H 8	1.97812	8.68	0.86	0.08
LP (2) O 42		σ^* C 33 – H 35	1.98233	10.	0.79	0.08
LP (2) O 42	1.92549	σ^* O 42 – C 50	1.98389	15.45	0.68	0.09
LP (2) O 42		σ^* O 42 – C 52	1.98722	57.08	0.54	0.16
LP (2) O 42		σ^* C 52 – H 58	1.98353	14.02	2.94	0.18
LP (2) O 46	1.88823	σ^* O 42 – C 52	1.98722	22.85	0.38	0.08
LP (1) O 47		σ^* C 24 – H 26	1.97962	12.17	1.02	0.10
LP (1) O 47		σ^* C 33 – H 34	1.98150	8.79	0.97	0.08
LP (1) O 47		σ^* C 33 – H 35	1.98233	20.73	0.95	0.13
LP (1) O 47		σ^* O 42 – C 50	1.98389	29.87	0.84	0.14
LP (1) O 47	1.97854	σ^* O 42 – C 52	1.98722	107.49	0.70	0.25
LP (1) O 47		σ^* O 47 – H 64	1.98835	7.23	1.08	0.08
LP (1) O 47		σ^* C 50 – H 56	1.97539	15.00	0.96	0.11
LP (1) O 47		σ^* C 52 – H 58	1.98353	28.32	3.10	0.27
LP (1) O 47		σ^* C 53 – H 60	1.97649	112.04	3.67	0.57
LP (2) O 47	1.94911	σ^* O 42 – C 52	1.98722	12.39	0.37	0.06
